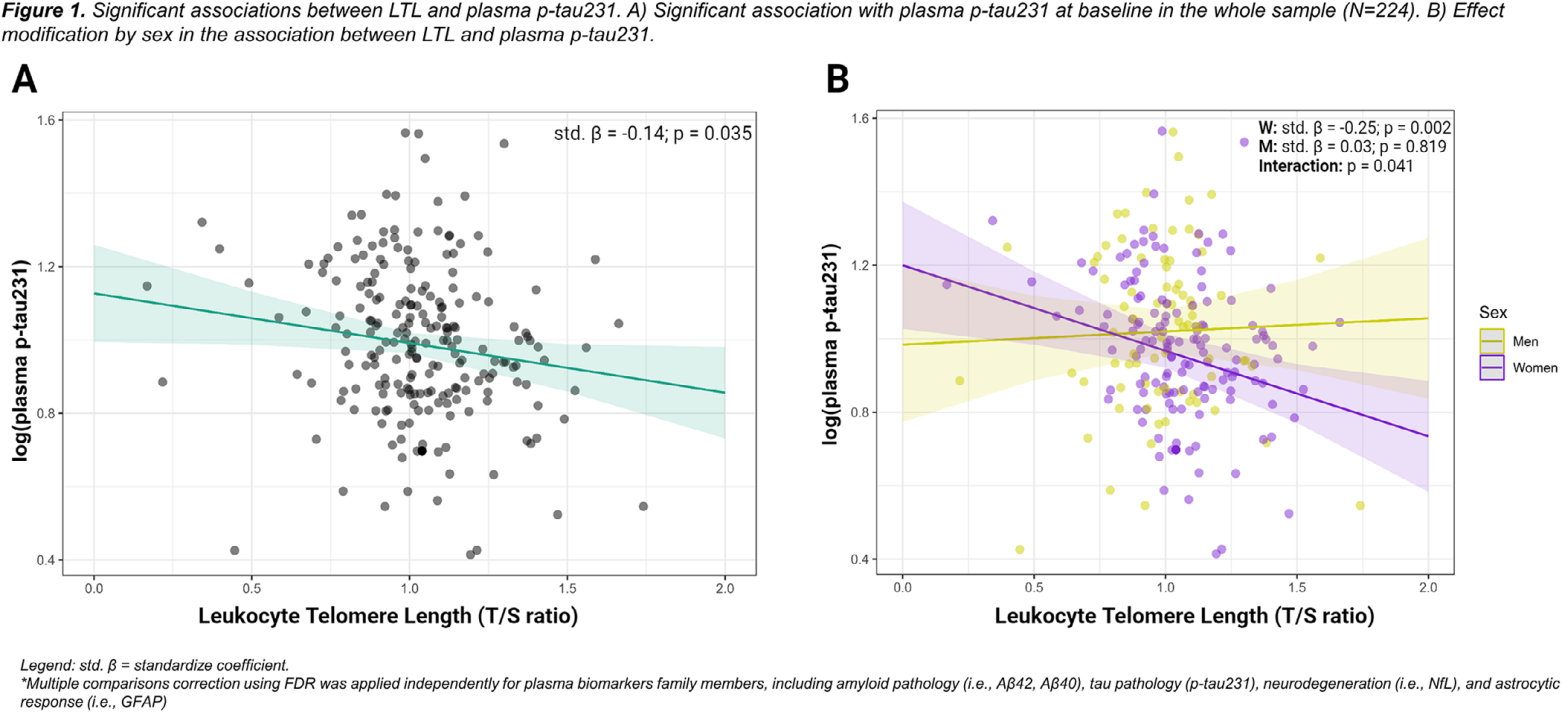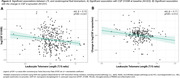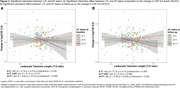# Longer leukocyte telomere length is associated with lower markers of early Alzheimer's disease pathology, astrocytic reactivity, and synaptic dysfunction

**DOI:** 10.1002/alz.091325

**Published:** 2025-01-09

**Authors:** Blanca Rodríguez‐Fernández, Armand González Escalante, Patricia Genius, Paula Ortiz‐Romero, Ann Brinkmalm, Carolina Minguillón, Gwendlyn Kollmorgen, Clara Quijano‐Rubio, Nicholas J. Ashton, Henrik Zetterberg, Kaj Blennow, Juan Domingo Gispert, Arcadi Navarro, Marc Suarez‐Calvet, Marta Crous‐Bou, Natalia Vilor‐Tejedor, for the ALFA study

**Affiliations:** ^1^ Barcelonaβeta Brain Research Center (BBRC), Pasqual Maragall Foundation, Barcelona Spain; ^2^ IMIM (Hospital del Mar Medical Research Institute), Barcelona Spain; ^3^ Centre for Genomic Regulation (CRG), Barcelona Institute of Science and Technology (BIST), Barcelona Spain; ^4^ Universitat Pompeu Fabra, Barcelona Spain; ^5^ Hospital del Mar Research Institute (IMIM), Barcelona Spain; ^6^ Clinical Neurochemistry Laboratory, Sahlgrenska University Hospital, Mölndal Sweden; ^7^ Barcelonaβeta Brain Research Center (BBRC), Barcelona Spain; ^8^ Centro de Investigación Biomédica en Red de Fragilidad y Envejecimiento Saludable (CIBERFES), Instituto de Salud Carlos III, Madrid Spain; ^9^ Roche Diagnostics GmbH, Penzberg Germany; ^10^ Roche Diagnostics International Ltd., Rotkreuz Switzerland; ^11^ Wallenberg Centre for Molecular and Translational Medicine, University of Gothenburg, Gothenburg Sweden; ^12^ Department of Psychiatry and Neurochemistry, Institute of Neuroscience and Physiology, The Sahlgrenska Academy, University of Gothenburg, Mölndal, Gothenburg Sweden; ^13^ King's College London, London UK; ^14^ NIHR Biomedical Research Centre for Mental Health and Biomedical Research Unit for Dementia at South London and Maudsley, NHS Foundation, London UK; ^15^ Wisconsin Alzheimer’s Disease Research Center, University of Wisconsin School of Medicine and Public Health, Madison, WI USA; ^16^ UK Dementia Research Institute, University College London, London UK; ^17^ Department of Psychiatry and Neurochemistry, Institute of Neuroscience and Physiology, The Sahlgrenska Academy at the University of Gothenburg, Mölndal Sweden; ^18^ Department of Molecular Neuroscience, UCL Institute of Neurology, London UK; ^19^ Hong Kong Center for Neurodegenerative Diseases, Clear Water Bay Hong Kong; ^20^ Centro de Investigación Biomédica en Red Bioingeniería, Biomateriales y Nanomedicina, Instituto de Salud Carlos III, Madrid Spain; ^21^ Centro Nacional de Investigaciones Cardiovasculares (CNIC), Madrid Spain; ^22^ Institute of Evolutionary Biology (CSIC‐UPF), Department of Experimental and Health Sciences, Universitat Pompeu Fabra, 08003, Barcelona Spain; ^23^ Institució Catalana de Recerca i Estudis Avançats (ICREA), Barcelona Spain; ^24^ Centro de Investigación Biomédica en Red de Fragilidad y Envejecimiento Saludable (CIBERFES), Madrid Spain; ^25^ Servei de Neurologia, Hospital del Mar, Barcelona Spain; ^26^ Department of Epidemiology, Harvard TH Chan School of Public Health, Boston, MA USA; ^27^ Catalan Institute of Oncology (ICO)‐Bellvitge Biomedical Research Center (IDIBELL), Hospitalet de Llobregat Spain; ^28^ Department of Clinical Genetics, Erasmus University Medical Center, Rotterdam Netherlands

## Abstract

**Background:**

Leukocyte telomere length (LTL) serves as a proxy for tissue‐specific TL and peripheral immune aging. Its association with aging‐related brain endophenotypes, cognitive functioning, and Alzheimer's disease (AD) risk is established, but the underlying molecular mechanisms remain elusive. Investigating LTL's association with AD biomarkers is crucial for identifying its role in brain resilience and disease progression.

**Method:**

We included middle‐aged adults at risk of AD dementia from the ALFA+ study. In CSF, we measured Aβ42, Aβ40, NfL, GFAP, α‐synuclein, neurogranin, S100B, sTREM2, YKL40, and IL6 using the exploratory Roche NeuroToolKit robust prototype assays; p‐tau181 was measured using Elecsys^®^; GAP‐43 using an in‐house ELISA; and Synaptotagmin‐1 using mass spectrometry. In plasma, we measured Aβ42, Aβ40, p‐tau231, NfL, and GFAP using Simoa. LTL was determined by qPCR from DNA extracted from peripheral blood leukocytes. Multiple linear regression models were used to evaluate the association between LTL and both baseline and the change in CSF and plasma biomarker levels over a three‐year period. Models accounted for age, sex and APOE‐ε4 status, and time between measurements, when interrogating the change in biomarkers. Sensitivity analyses included models adjusted by CSF Aβ42/40, CSF p‐tau181. Sex‐, APOE‐ε4 and disease‐specific effects were explored through interaction and stratified models.

**Result:**

In plasma, longer LTL was associated with lower plasma p‐tau231 at baseline [Figure 1A]. This association was mainly driven by women [Figure 1B]. In CSF, longer LTL was associated with reduced S100B at baseline [Figure 2A] and decreased α‐synuclein over time [Figure 2B], independently of CSF Aβ42/40 and p‐tau181 levels. Over time, longer LTL was associated with increasing CSF IL6 levels among A+T‐ individuals, while the opposite direction was observed among A+T+ individuals [Figure 3A‐3B].

**Conclusion:**

Longer LTL was associated with lower plasma p‐tau231, an early marker of AD pathology, specifically in women. Furthermore, longer LTL was independently associated with lower biomarkers of astrocytic and synaptic dysfunction, regardless of AD pathology. Nevertheless, an AT‐dependent shift in LTL's impact on CSF IL6 change emerged over time. These results collectively propose that peripheral immune aging could be related to the central nervous system along the AD continuum, either independently or in a sex‐ and disease‐specific manner.